# Fingolimod in active multiple sclerosis: an impressive decrease in Gd-enhancing lesions

**DOI:** 10.1186/s12883-014-0164-5

**Published:** 2014-08-20

**Authors:** Anne-Hilde Muris, Linda Rolf, Jan Damoiseaux, Ellen Koeman, Raymond Hupperts

**Affiliations:** 1 School for Mental Health and Neuroscience, Maastricht University Medical Center, Universiteitssingel 40, Maastricht, theNetherlands; 2 Academic MS Center Limburg, Orbis Medical Center, Sittard, the Netherlands; 3 Central Diagnostic Laboratory, Maastricht University Medical Center, Maastricht, the Netherlands; 4 Department of Radiology, VU University Medical Center, Amsterdam, the Netherlands; 5 Postal address: Maastricht University Medical Center, Central Diagnostics Laboratory - RVE Laboratories and Imaging, Maastricht, 6202 AZ, the Netherlands

**Keywords:** Disease modifying therapies, Fingolimod, Multiple sclerosis, MRI, Relapsing remitting, T1gadolinium enhancing lesions, T2 lesions

## Abstract

**Background:**

Fingolimod is a disease modifying therapy (DMT) in highly active relapsing remitting multiple sclerosis (RRMS), as is natalizumab. Fingolimod decreases annual relapse rates and gadolinium enhancing lesions on MRI as compared to either interferon beta (IFNβ) or placebo. The effect of fingolimod on MRI outcomes compared to natalizumab treatment has not been investigated in (head to head) clinical trials. Clinical experience with natalizumab is much more extended and in general practice often preferred.

**Case presentation:**

This case describes a 31-year old woman with RRMS, who experienced severe side effects on natalizumab. After a voluntary four months treatment free period, a severe relapse appeared which was treated with prednisone and plasmapheresis; thereafter fingolimod was initiated. In the following months MRI signs improved spectacularly.

**Conclusion:**

This case suggests that fingolimod might be a good alternative for natalizumab, especially for use in RRMS patients, with highly active, advanced disease, when natalizumab treatment is stopped due to side effects or even after a severe relapse.

## Background

Fingolimod (FTY720, Gilenya®, Novartis Pharma AG, Basel, Switzerland) is like natalizumab (Tysabri®, Biogen Idec Inc, Weston, MA, USA) a single disease modifying therapy (DMT) in highly active relapsing remitting multiple sclerosis (RRMS) patients. Fingolimod is registered in 80 countries across the world. In some countries, like the USA, Switzerland, Australia and Russia, fingolimod is approved as a first line treatment while in Europe and Canada fingolimod is a second line therapy especially for those patients who are non-respondent to at least one other DMT like interferon beta (IFNβ) or glatiramer acetate (GA) or who have rapidly evolving MS [1]–[3].

Fingolimod is an oral sphingosine 1-phosphate receptor modulator and acts as a functional antagonist reducing the amount of circulating pathogenic lymphocytes by inhibiting mainly naïve T cells and central memory T cells to egress from the lymph nodes. It might also play a role in the neuroprotection of the central nervous system (CNS) [4]. Phase II and phase III studies with fingolimod have shown a decrease in annual relapse rate, as well as a reasonable decline in gadolinium (Gd) enhancing lesions on MRI, both in number and volume, after up to 36 months of fingolimod treatment compared to either first line treatment with IFNβ or placebo [5]–[7].

The effect of fingolimod compared to natalizumab treatment has never been investigated in a head-to-head clinical trial. However, natalizumab was approved approximately five years before fingolimod and therefore the clinical experience with natalizumab is much more extended and in general practice often preferred [1],[2],[8]. When natalizumab is discontinued, because of various reasons, a switch to fingolimod is an obvious next step. However, reactivation of disease in patients switching from natalizumab to fingolimod is reported in a considerable proportion of patients [9]–[11].

Here we describe a case of a patient who suffered from highly active RRMS which was treated with fingolimod following a severe relapse after discontinuation of natalizumab and a treatment free interval of four months. We consider this case as a striking example of the positive effect that fingolimod treatment may have especially on MRI outcome, even after successful natalizumab treatment.

## Case presentation

A 31-year old woman was diagnosed with RRMS at the age of 25. Three years before diagnosis she presented with a first event of one-sided optic neuritis. She did not have any further medical history.

**Figure 1:**
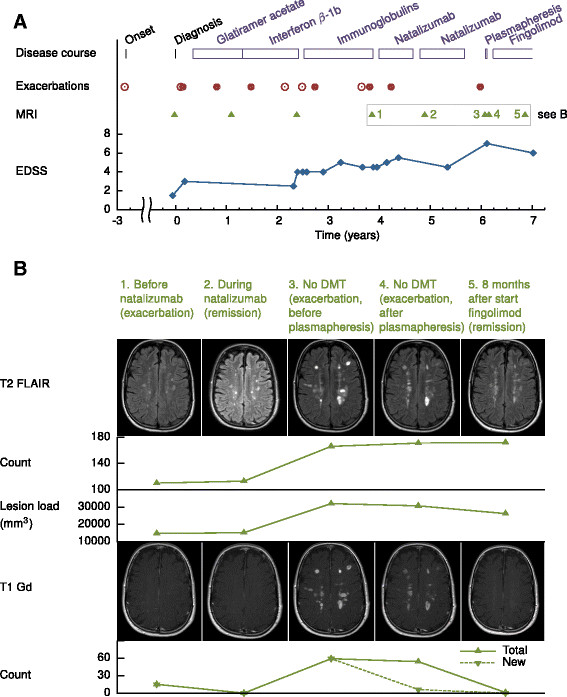
**Schematic overview of disease course. (A)** Disease course from diagnosis, including **(B)** quantification of MRI (T1gado, T2 and T2 FLAIR) before and after start of fingolimod. Shown are patient’s treatment regime, relapses (in closed dots when treated with methylprednisolone (MP), in open dots when untreated), time points of all MRI and EDSS scores. The lower part of the figure **(B)** shows the last five, most relevant, subsequent T2 FLAIR and T1 Gd MRI’s. T2 lesion count and lesion load (measured using conventional T2 MRI and FLAIR MRI) and T1 Gd lesion counts are shown. T2 lesion count and lesion load were quantified by an expert reader in MIPAV (version 5.1.1, Center for Information Technology, Bethesda, Maryland). At follow up visits subtracted images were used for MRI analyses. Total T2 lesion load at follow up was calculated as the lesion load at baseline (MRI 1) plus negative and/or positive activity change. Time points of MRI in MS course: MRI 1 – before start of natalizumab treatment (during exacerbation). MRI 2 – just after restart natalizumab treatment (remission). MRI 3 – during exacerbation 4 months after natalizumab discontinuation before plasmapheresis. MRI 4 – during exacerbation 4 months after natalizumab discontinuation after plasmapheresis. MRI 5 – 8 months after start of fingolimod (remission). Abbreviations: DMT: disease modifying therapy; EDSS: Expanded Disability Status Scale; FLAIR: Fluid Attenuation Inversion Recovery.

Several first line treatments, i.e. GA and IFNβ-1b had insufficient effect: exacerbation rate remained high and MRI showed a slight increase in lesion number (Figure 1A). While second line therapy was not indicated because of patient’s desire to become pregnant, treatment with intravenous immunoglobulins was initiated. Immunoglobulins are not a registered therapy in MS, but can be used off-label if no other options are available [12]. However, relapse rate remained high and one and a half year after IFNβ-1b was stopped, she was still in a moderate clinical condition and MRI showed multiple new T1 Gd enhancing lesions. Therefore, after a third relapse during immunoglobulin treatment, treatment with natalizumab was initiated. The one relapse she experienced during the natalizumab treatment was in an early phase, and therefore might have been still the result of the highly active MS before the effects of natalizumab. MRI, 11 months after initiation of natalizumab, showed a slight increase in white matter lesions on T2 (FLAIR) MRI without any T1 Gd enhancing lesions (Figure 1B). At a later stage the patient was tested positive for anti-JC virus antibodies and suffered from severe side effects, like frequent urinary tract infections and herpes zoster infections. All together this made discontinuation of natalizumab after 20 months of treatment inevitable. After a voluntary treatment-free interval of four months, she had a serious relapse with right sided hemiplegia, problems with coordination, ataxia and dizziness, for which an acute admission into the hospital was needed. Tests for JC-virus DNA in CSF were negative, excluding progressive multifocal leucoencephalopathy (PML), but MRI of the brain showed an increased number of T2 lesions on conventional T2 MRI, an increased volume on T2 FLAIR MRI and an increased number of T1 Gd enhancing lesions throughout the white matter (Figure 1B). After plasmapheresis and methylprednisolone (MP) treatment, control MRI showed only minor improvement. At that time fingolimod treatment was started. From that moment on the patient’s condition gradually improved and she remained relapse-free. Moreover, most recent MRI of the brain (8 months after the initiation of fingolimod) showed a striking decrease in the number of T1 Gd enhancing white matter lesions (Figure 1A and B), without any new Gd enhancing lesions.

Natalizumab and fingolimod both are registered immunomodulatory therapies in RRMS, currently known to have comparable effectiveness. Natalizumab, in general practice frequently used, results in clinical and MRI stabilization, or even improvement [13]. However, in the long term, natalizumab treatment has some shortcomings. Side effects like frequent urinary tract infections or herpes infections can occur. Also the increasing risk of getting PML in anti-JC virus antibody positive patients can lead to discontinuation of treatment. Fingolimod, with a different mechanism of action but shown to be also highly effective in reducing relapse rate in RRMS, might therefore be a good alternative for natalizumab [1],[14].

A potential risk of natalizumab discontinuation is the risk of reactivation of disease, as is also described in our case presentation. Radiological and clinical rebound, in which disease activity increases to levels even higher than baseline, has been described between 1 and 6 months after discontinuation of natalizumab [15]. However, in most cases disease activity returns to baseline with a peak four months after withdrawal [16]. Fingolimod has been described to potentially mitigate the reactivation of disease after withdrawal of natalizumab [17]. However, severe relapses in the first months after switching from natalizumab to fingolimod have also been reported [9]–[11]. These differences in outcome of fingolimod treatment used to overcome disease reactivation might be due to differences in duration of the wash out period of natalizumab. The wash out period between natalizumab and fingolimod is considered not to exceed two or three months [18],[19]. On the other hand, recently an observational study showed that relapses after switching from natalizumab to fingolimod occurred independently of the wash-out period [20].

In this case presentation, fingolimod was not used to prevent a rebound effect or reactivation of disease after discontinuation of natalizumab. Instead, after natalizumab withdrawal initially the patient did not receive any immunomodulatory medication. Only after the severe relapse, four months later, fingolimod was started. Afterwards, the patient stabilized clinically and T1 Gd enhancing lesions decreased spectacularly with only one persistent Gd lesion and no new Gd enhancing lesions after 8 months (Figure 1B). Although, Gd enhancing lesions may become inactive after 2–3 months, this decrease from 54 T1 Gd enhancing lesions to only one persistent is conspicuous and a treatment effect of fingolimod therefore almost undeniably.

## Conclusions

This case shows and confirms that fingolimod might be radiologically and clinically as effective as and a good alternative for natalizumab in highly active advanced RRMS or possibly even in patients developing relapsing progressive MS. Based on this case report one might speculate fingolimod to be a good alternative for natalizumab in anti JC virus positive patients. Moreover, it might even be useful in the treatment regime of a MS patient after a severe relapse.

## Consent

Written informed consent was obtained from the patient for publication of this case report and any accompanying images. A copy of the written consent is available for review by the Editor of this journal.

## Competing interests

AM, LR, JD, EK declare that there is no conflict of interest. RH received honoraria for lectures and advisory boards and Research Grants from Merck, Biogen-Idec, Sanofi-Genzyme, Novartis and TEVA.

## Authors’ contributions

Primary patient care and patient recruitment: RH. Manuscript drafting: AM and LR. Quantification of MRI data: EK. Critical revision of the manuscript: AM, LR, JD, EK and RH. All authors read and approved the final manuscript.
